# Changes in Life Situations during the SARS-CoV-2 Virus Pandemic and Their Impact on Eating Behaviors for Residents of Europe, Australia as Well as North and South America

**DOI:** 10.3390/nu13103570

**Published:** 2021-10-12

**Authors:** Paulina Górska, Ilona Górna, Izabela Miechowicz, Juliusz Przysławski

**Affiliations:** 1 Department of Bromatology, Poznań University of Medical Sciences, 60-354 Poznań, Poland; igorna@ump.edu.pl (I.G.); jprzysla@ump.edu.pl (J.P.); 2 Department of Computer Science and Statistics, Poznań University of Medical Sciences, 60-806 Poznań, Poland; iza@ump.edu.pl

**Keywords:** COVID-19, eating behaviors, life situations

## Abstract

Many people’s life situations are changing as a result of restrictions being imposed by national governments to limit the spread of the virus. These may be associated with additional factors (emotional or financial, for example) that influence eating behavior and physical activity levels. Therefore, the aim of this study was to show whether there is a relationship between a changing life situation during the pandemic and eating behavior as well as physical activity. An online survey was conducted between 28 April and 16 July 2020 with 921 participants from European countries and countries outside Europe (South and North America, Australia). An analysis of the obtained results showed an unfavorable relationship between a change in life situation during the pandemic and eating behavior as well as physical activity. This was observed mainly among students who returned to their family homes and respondents whose working hours increased. Students were more likely to snack between meals (51.13%, *p <* 0.001) and to consume more sweets (45.11%, *p <* 0.001) and savory snacks (30.83%, *p <* 0.001). Those whose working hours had increased, consumed morefast foods (13.57%, *p <* 0.05) during that time. On the other hand, the study results indicated that a change in life situation during the pandemic can also have a positive impact on eating behavior and physical activity. This was exhibited by individuals who transitioned to remote working. An improvement in the regularity of eating (38.86%, *p <* 0.001) was recorded for this group. The relationship between a change in life situation and eating behavior was further emphasized by the fact that people whose life situation had not changed were more likely to declare no change in the regularity of eating (62.86%, *p <* 0.001) and snacking (61.71%, *p <* 0.001). At the same time, they were less likely to exhibit a higher intake of sweets (22.29%, *p <* 0.01) and salty snacks (13.14%, *p <* 0.01). The study results indicated that a change in the nutritional situation during the pandemic may have had both negative and positive effects on eating behavior and physical activity. Finding these relationships may help identify groups that are particularly vulnerable to reduced diet quality and reduced levels of physical activity. Considering the immunomodulating effects of diets and the fact that physical activity is essential for maintaining good health, further research in this area is needed.

## 1. Introduction

In early January 2020, the Chinese Center for Disease Control and Prevention (CDC) identified a new coronavirus: SARS-CoV-2. Infections with the new virus led to the development of a disease known as “COVID-19”. A few months after the new coronavirus was identified, the World Health Organization (WHO) declared COVID-19 a pandemic [1,2].

Social distancing policies were among the most effective of the various preventive measures used to control the spread of the virus. Results of studies conducted during the SARS-CoV outbreak show that, despite their effectiveness, these measures can also have negative consequences. A higher risk of developing depression, post-traumatic stress disorder, feeling of exhaustion, anger, and sleep disorders are all associated with social distancing policies and have an impact on diet quality [3].

Adverse effects of the pandemic on eating habits and physical activity may be exacerbated by changes in an individual’s life situation (e.g., the need to change to remote studying or working, 24-h childcare, reduced income or loss of job). Ingram et al. (2020) reported that people who lost their jobs during the pandemic were more likely to exhibit unhealthy eating habits [4]. They also noted higher alcohol consumption among respondents with 24-h childcare during this period. Phillipou et al. (2020), on the other hand, observed that physical activity levels decreased for individuals who switched to remote work during the pandemic [5]. Changing the mode of work or study (in the case of students) to remote requires more time spent at home. The study results by Nishijima et al. (2020) showed that more respondents in the group with extended at-home hours experienced an increase or decrease in total physical activity and eating behavior (snacking, food intake, alcohol drinking) [6]. Constant et al. (2020) observed that the level of physical activity during the pandemic was declining. Their research showed that factors such as male sex, living in urban density, having a garden, financial difficulties, and lack of fear control negative influences. On the other hand, the results of the study by Constant et al. (2020) showed that factors can positively affect eating behavior during a pandemic. These include factors like living with more than two persons, perceived efficacy, and having a terrace [7]. The research conducted by De Backer et al. (2020) showed that emotional factors may have a more significant influence on eating behavior in women, while in men, financial factors are more important [8]. Similar conclusions were drawn by Stanton et al. (2020), who observed that women are more prone to depression and anxiety during a pandemic, which translates into a change in lifestyle (including lower levels of physical activity, higher alcohol consumption). The extent to which emotional factors influence a lifestyle change may depend not only on gender but also on body weight [9]. Flanagan et al. (2020) showed that the increase in anxiety scores is more pronounced in people with obesity [10].

Much research on changes to eating behaviors and/or physical activity during the pandemic considers differences such as gender or age, for example. We already presented such a relationship in a previously published study on 279 residents of European countries (Poland, England and Scotland, Spain, Portugal, Italy). It aimed to demonstrate whether country, gender, age, and place of residence (rural or urban) impacted changes to eating behaviors and activity levels. In this publication, we present the results for a larger group of 921 people living not only in Europe but also in Australia and North and South America. Furthermore, the aim was not to show a relationship between place of residence, gender, and age and changes in eating behaviors and physical activity levels. The findings presented in this article address a different issue: the impact of changing life situations during the pandemic on changes in eating behaviors and physical activity levels. They attempt to answer the question of how a reduction in business income, round-the-clock childcare, transition to remote work, changes in working hours, and a return to the family home (for students) might affect eating behaviors and physical activity levels during the pandemic.

## 2. Materials and Methods

### 2.1. Study Design

An online survey was conducted between 28 April and 16 July 2020 to investigate the relationship between a change in life situation during the SARS-CoV-2 pandemic and eating behaviors and physical activity. A specially devised, anonymous questionnaire was used as the research tool. In addition to the Polish version of the questionnaire, English, Italian, Spanish, and Portuguese versions were prepared in cooperation with translators. The snowball sampling method was used to recruit respondents for the study. Researchers and study participants shared the questionnaire via social media (e.g., Facebook, mainly on groups for people living in a particular country, e.g., Poland, England, the United States; Instagram, Twitter, LinkedIn). The online survey was conducted in accordance with national and international legislation and the Declaration of Helsinki. The K. Marcinkowski Medical University, in Poznań, bioethics committee confirmed the research does not exhibit traits of a medical experiment. All participants were made aware of the aims and requirements of the study. They completed a questionnaire using Google Forms and were not asked for personal data. The responses were then downloaded as a Microsoft Excel spreadsheet, which ensured their anonymity.

### 2.2. Participants

There were 921 people (79.70% women and 20.30% men) aged 18 and over who took part in the survey. People living in Europe, as well as South America, North America, and Australia, took part in the study. Respondents who answered the questionnaire did not have to be nationals of the country in question but resided there during the pandemic. Study participants had to be more than 18 years old. A question in the questionnaire verified this.

### 2.3. Measures

The survey questionnaire was made up of sections on sociodemographic data (gender, age, place of residence), eating behaviors, physical activity, the impact of the pandemic on the ease of following a weight loss diet started before the pandemic, and change in the living situation during the pandemic. In the section on eating behaviors, respondents were asked about changes to the regularity of their meals, snacking, and frequency of consumption of products with low nutritional values (sweets, savory snacks, fast food, fried food), alcohol, milk and dairy products, cereal products, eggs, meat, fish, fats (butter, vegetable oils, margarine), vegetables, fruit, and pulses. Research subjects were also asked about changes to physical activity levels during the pandemic. Questions were structured to allow a subjective assessment of changes in this area compared to a pre-pandemic period. To that end, they included options such as “less/more”, “more frequently/less frequently”, or “no changes”. The questionnaire also included a question about changes to one’s life situation during the pandemic. Respondents could select responses such as “reduction in business income”, “24/7 childcare”, “transition to remote work”, “increased working hours”, “reduced working hours”, “returning to the family home” (for students) and “the pandemic has not affected any aspect of my life”.

### 2.4. Statistical Analyses

Calculations were performed using StatSoft’s Statistica v. 12 software and Cytel’s StatXact v. 9.0.0. An α = 0.05 significance level was adopted. Results were considered to be statistically significant for *p <* α. The Shapiro–Wilk test was used to test the distribution of variables for normality. To compare two groups of normally distributed variables with the same variance, the Student’s *t*-test was used for unrelated individuals. The Mann–Whitney test was used where the variables were not normally distributed. To test for dependencies among categorical variables, the Chi-squared test for independence, Fisher’s exact test, or the Fisher–Freeman–Halton was applied. In order to verify whether the indicated relationships did not result from the relationship between sociodemographic factors and pandemic factors, a log-linear analysis was performed. The statistical significance of the analyzed interactions was tested with the Pearson chi-square test.

## 3. Results

### 3.1. Sociodemographic Data and Information on Changes to Life Situations

There were 921 individuals who took part in the study. The general characteristics of the study population are shown in Table 1. In the entire sample, 38.00% (n = 350) transitioned to remote work, 15.20% (n = 140) increased and 16.94% (n = 156) decreased their working hours, 14.44% (n = 133) returned to the family home (students), 11.62% (n = 107) reported a reduction in business income during the pandemic, 11.29% (n = 104) had to provide 24/7 childcare, and for 19.00% (n = 175) the life situation during the pandemic did not change.

Considering the sociodemographic factors, the relationship between gender, age, country, and the change in life situation was made. Its results showed that the most significant differences occurred depending on age. There were statistically significant differences between the respondents <35 years and >35 years, and reduced business income (*p* < 0.05) and 24/7 childcare, transition to remote work, increased working hours, return to the family home (student), and the answer whose respondents declared that their life situation had not changed (*p* < 0.01). The results are shown in Table 2.

### 3.2. Meal Consumption Regularity

The regularity of eating meals increased among 41.46% of people whose working hours decreased during the pandemic. Such a change was observed in 31.70% (*p <* 0.01) of the other respondents. The work mode was also significant, with 38.86% of those who switched to remote working indicating that the regularity of their meals had improved. The same was true for was 28.72% (*p <* 0.01) of those who did not change their work mode. Another factor with a positive impact on meal regularity was a return to the family home for students. Increased regularity was declared by 46.62% of students compared to 30.20% among other respondents (*p <* 0.001).

For people whose life situations did not change during the pandemic, no change in meal regularity was observed by 62.86%. Of the sample, 42.63% (*p <* 0.001) stated that their life situations had changed. The results are shown in Table 3.

In order to check whether the indicated relationships did not result from the relationship between sociodemographic factors, a log-linear analysis was performed. The results showed that there was still a significant statistical relationship between the regularity of consuming meals and transition to remote work and decreased working hours (*p <* 0.01), return to the family home (students) (*p <* 0.05), and no change in life situations during a pandemic (*p <* 0.001).

### 3.3. Snacking

More than half (51.13%) of the students who returned to the family home declared that they started snacking between meals (*p <* 0.001). Those whose life situation did not change during the pandemic mostly (61.71%) said that the regularity of their meals did not change either. Respondents whose life situation had changed accounted for 42.36% (*p <* 0.0001). The results are shown in Table 4.

In order to check whether the indicated relationships did not result from the relationship between sociodemographic factors, a log-linear analysis was performed. The results showed that there was still a significant statistical relationship between snacking and return to the family home (students) (<0.01) and no change in life situations during a pandemic (*p <* 0.001).

### 3.4. Eating Behaviours

#### 3.4.1. Sweets

Those whose working hours increased during the pandemic were more likely than others to indicate an increased consumption of sweets: 39.29% compared to 29.19% among other respondents (*p <* 0.01). A similar relationship was observed for students who returned to their family homes. Of them, 45.11% said they consumed more sweets, compared to 22.29% of those whose living situation had not changed in this respect (*p <* 0.001). On the other hand, for people whose life situation had not changed, a smaller proportion (22.29%) declared higher sweet consumption than among the remaining respondents, 32.71% (*p <* 0.01).

To check whether the indicated relationships did not result from the relationship between sociodemographic factors, a log-linear analysis was performed. The results showed that there was still a significant statistical relationship between increased consumption of sweets and return to the family home (students) (<0.001) and no change in life situations during a pandemic (*p <* 0.05). However, it was observed that, after taking into account the influence of sociodemographic factors, the relationship between increased working hours and consumption of sweets was on the verge of statistical significance (*p =* 0.05).

#### 3.4.2. Savory Snacks

Students who returned to their family homes during the pandemic were more likely than others to report increased consumption of savory snacks (30.83% vs. 19.04%, *p <* 0.001). On the other hand, for respondents whose life situation had not changed, the percentage who increased their consumption of savory snacks was lower compared to the others (22.52%) at 13.14% (*p <* 0.01).

To check whether the indicated relationships did not result from the relationship between sociodemographic factors, a log-linear analysis was performed. The results showed that there was still a significant statistical relationship between increased consumption of savory snacks and return to the family home (students) (<0.05) and no change in life situations during a pandemic (*p <* 0.05).

#### 3.4.3. Fast Food and Fried Food

Those whose working hours increased during the pandemic were more likely than others to report increased consumption of fast foods (13.57% vs. 8.32%, *p <* 0.05).

In the group of students who returned to their family home during the pandemic, 18.8% said they ate more fried foods. Among the other respondents, 8.25% declared such an increased consumption (*p <* 0.001).

To check whether the indicated relationships did not result from the relationship between sociodemographic factors, a log-linear analysis was performed. The results showed that there was still a significant statistical relationship between increased consumption of fast food and increased working hours (<0.05) but it was no longer statistically significant among students.

#### 3.4.4. Alcohol

Higher alcohol consumption during the pandemic was observed for people who had 24/7 childcare due to the closure of nurseries, preschools, and schools. A change in this area was declared by 25.00% of people in this category compared to 15.91% of other respondents (*p <* 0.01). A similar trend was seen for respondents whose working hours had decreased during the pandemic, with 22.44% saying they consumed more alcohol. For those whose working hours did not decrease this figure was 15.82% (*p <* 0.05).

In order to check whether the indicated relationships did not result from the relationship between sociodemographic factors, a log-linear analysis was performed. The results showed that there was still a significant statistical relationship between higher alcohol consumption and 24/7 childcare (<0.001) and decreased working hours (*p <* 0.05).

#### 3.4.5. Observing Healthy Eating Principles

Those who had 24/7 childcare were less likely than others to declare that they adhered more stringently to healthy diets during the pandemic. A change in this area was declared by 12.50% of respondents in this category. For the others, the figure was 87.5% (*p <* 0.01).

To check whether the indicated relationships did not result from the relationship between sociodemographic factors, a log-linear analysis was performed. It was observed that, after taking into account the influence of sociodemographic factors, the relationship between 24/7 childcare and healthy eating principles was on the verge of statistical significance (*p =* 0.05).

#### 3.4.6. Observing Weight Loss Diets Started before the Pandemic

Respondents were asked whether during the pandemic they found it easier, more difficult, or the same to follow a weight loss diet started before the pandemic. A statistically significant relationship in this respect was noted for those who transitioned to remote work. Of these, 11.75% indicated that following a weight loss diet was easier compared to 8.96% of respondents not in this category (*p <* 0.05). In contrast, a smaller proportion of students who returned to their family home reported a positive impact: 9.77% compared to 10.06% of those for whom the situation was the same (*p <* 0.05).

In order to check whether the indicated relationships did not result from the relationship between sociodemographic factors, a log-linear analysis was performed. It was observed that, after taking into account the influence of sociodemographic factors, the relationship between transition to remote work and following the rules of a weight-loss diet was on the verge of statistical significance (*p =* 0.05). It was also reported that the relationship between returning to the family home and following the rules of a weight-loss diet was no longer statistically significant among students.

### 3.5. Consumption Frequency for Selected Product Groups

Research results indicated that the consumption frequency of the selected product groups may be affected by factors such as a reduction in business income, transition to remote work, reduction in working hours, and a return to the family home for students.

Respondents who reported a reduction in business income during the pandemic were more likely to observe a decrease in fat consumption (21.50% vs. 11.30%, *p <* 0.01), as well as an increase in the consumption of pulses (21.50% vs. 15.85%, *p <* 0.05).

Those who switched to remote work during the pandemic were more likely than others to report increased consumption of eggs (29.43% vs. 21.02%, *p <* 0.01) and meat (20.86% vs. 11.73%, *p <* 0.001).

The frequency of consumption was also affected by a reduction in working hours. Those in that category during the pandemic were more likely to report lower consumption of fish (23.08% vs. 18.69%, *p =* 0.0374) and fats (18.59% vs. 11.24, *p =* 0.0354).

Changes to the consumption of specific food groups were also observed by students who returned to their family homes during the pandemic. Compared to the other respondents, they were more likely to declare an increase in the consumption of milk (31.58% vs. 17.89%, *p <* 0.001), meat (27.82% vs. 13.07%, *p <* 0.001), and fats (18.05% vs. 8.88%, *p <* 0.01).

Respondents whose life situations had not changed during the pandemic were more likely than others to declare that the frequency of consumption of selected product groups had remained the same. This was true for cereals (71.43% vs. 62.6%, *p <* 0.01), meat (77.71% vs. 65.15%, *p <* 0.01), eggs (73.71% vs. 66.22%, *p <* 0.05), and fruit (63.43% vs. 53.00%, *p <* 0.05).

No statistically significant relationship was observed with the frequency of consumption of the different product groups for those who indicated that their life situation had changed in terms of 24/7 childcare and increased working hours (*p* > 0.05).

In order to check whether the indicated relationships did not result from the relationship between sociodemographic factors, a log-linear analysis was performed. The results showed that there was still a significant statistical relationship between consumption of fats and reduced business income (*p <* 0.01), decreased working hours (*p <* 0.01), and return to the family home (*p <* 0.01). There was also still a statistically significant relationship between egg consumption and transition to remote work (*p <* 0.01), but the case of no change in life situations during the pandemic was already on the verge of statistical significance (*p =* 0.05). After taking into account the sociodemographic factors, there was still a statistically significant difference in the case of meat consumption and increased working hours (*p <* 0.01), return to the family home (students) (*p <* 0.001), and no change in life situations during the pandemic (*p <* 0.05). Further, there was also a statistically significant relationship between reduced business income and pulses consumption (*p <* 0.01), return to the family home (students) and milk consumption (*p <* 0.001) and no change in life situations during the pandemic and cereals consumption (*p <* 0.05). However, after log-linear analysis, the relationship between fish consumption and decreased working hours was almost statistically significant (*p* = 0.06), as was the relationship between no change in life situations during the pandemic and fruit consumption (*p* = 0.09).

### 3.6. Physical Activity

Students who returned to their family homes during the pandemic were more likely than other respondents to declare increased physical activity levels (*p <* 0.01). In contrast, those whose life situation had not changed were more likely than the others to state that their physical activity had remained the same (*p <* 0.001). The results are shown in Table 5.

A log-linear analysis was performed to check whether the indicated relationships did not result from the relationship between sociodemographic factors. It was observed that, after taking into account the influence of sociodemographic factors, the relationship between no change in life situations during the pandemic and the level of physical activity was still statistically significant (*p <* 0.01). However, the relationship between return to the family home (students) and the level of physical activity was on the verge of statistical significance (*p =* 0.05).

## 4. Discussion

### 4.1. The Extent of Observed Changes in Life Situations

The study results showed that the extent of changes in life during a pandemic may depend on factors such as age, gender, and place of residence. Inhabitants of European countries more often indicated reduced business income. One of the reasons for this may be the difference in the number of people who run their own business in European and non-European countries. Another reason may be differences in the introduced restrictions (e.g., closing restaurants, limiting the operation of shops).

Changes also depend on gender: Women more often indicated that they looked after children during the pandemic and their working hours decreased. This could lead to an increased level of stress related to round-the-clock childcare, as well as a worse financial situation.

However, the greatest number of differences was observed between people under and over 35 years. Younger people more often declared that their working time had increased. This could be because some of them had no children. Therefore, the closure of kindergartens and schools did not necessitate 24-h childcare. Perhaps they had to take over the responsibilities of older workers who went on vacation for this reason. It was also observed that younger people were more likely to switch to remote work. As the study showed, this may have had a positive impact on the regularity of food consumption. Other authors also observed positive changes in eating behavior after switching to remote working mode [11,12]. However, it should be borne in mind that remote work is usually sedentary. Therefore, perhaps it is worth considering the need to promote regular physical activity in this group.

### 4.2. The Relationship between Changes in Life Situation and Changes in Lifestyle

#### 4.2.1. Returning to the Family Home

Returning to the family home could have had a positive effect on eating behavior and physical activity due to the emotional support of the family and the change in living conditions. In this case, students who lived with their parents could be in a better position than those who lived alone. The study results showed that returning to the family home during the pandemic had a positive effect on the meal regularity and the level of physical activity. After considering sociodemographic factors, the relationship between returning to the family home and the level of physical activity was on the verge of statistical significance (*p =* 0.05). Therefore, in this case, other factors such as gender or place of residence could be decisive. According to Donati Zepp et al. (2020), increased physical activity is associated with healthier eating habits for young people [13]. This may be due to better appetite control, better food choices, and a reduced risk of emotional eating [13,14,15]. However, the study results showed that increasing levels of physical activity were not always associated with healthier eating habits. Negative changes were observed in higher consumption of sweets, savory snacks, and fats and more frequent snacking.

The negative impact of the pandemic on students’ eating behaviors may be due to higher levels of anxiety, stress, and depression observed among 18–30-year-olds [16,17]. Students belong to one of the groups whose lifestyle changed the most during the pandemic. The higher level of stress, anxiety, and depression among students could have been caused by limited contact with peers by changing the learning mode to remote learning and closing dormitories, as well as the inability to participate in numerous events. Moreover, a study of 52,000 Chinese showed that respondents in the 18–30 age group obtained the highest post-traumatic distress index scores [18]. It is thought that this was related to the increased amount of information (including false information) that they were exposed to via the media. In addition, the media are a source of food advertising, and increased exposure to that can influence dietary choices. In addition, the need to adapt to remote learning may also be an additional psychological burden that has a negative impact on eating behaviors [19,20]. On the other hand, some studies, such as the one by Di Renzo et al. (2020), showed that during the pandemic the diet of 18–30-year-olds was most similar to the Mediterranean diet, which is considered to be one of the healthiest known diets. However, the authors did not consider the fact that life situations changed due to the pandemic [21]. Therefore, it is not known whether there was a difference between the respondents who returned to the family home and the rest of the people. However, contrary to the results of our study, Zurita-Ortega et al. (2018) showed that people living in the family home had healthier eating habits. However, that research was not conducted during the pandemic. Therefore, one may assume that the return to a family home itself was important during the pandemic and the emotional factors associated with this specific period [22].

#### 4.2.2. Transition to Remote Work

Those who transitioned to remote work showed an improvement in meal consumption regularity, as well as greater ease in adhering to a weight loss diet started before the pandemic. A factor that might have been crucial here was the less frequent eating out when working remotely. In a systematic review, Lachat et al. (2012) found that more frequent eating out was associated with higher consumption of saturated fatty acids [11]. Llanaj et al. (2018) came to similar conclusions. They observed that eating meals out was conducive to higher consumption of sweets and soft drinks and contributed to a reduction in the consumption of fruit and vegetables [12].

After considering the sociodemographic factors, it was observed that the relationship between the change of working mode to remote work and adherence to the principles of a weight loss diet was at the border of statistical significance (*p =* 0.05). Therefore, it can be assumed that such factors as gender, place of residence, and age also played a significant role.

#### 4.2.3. Time as a Factor Influencing a Change in Lifestyle

Respondents whose working hours had increased reported higher consumption of fast food and sweets. On the other hand, those whose working time decreased more often declared eating meals more regularly during the pandemic.

Adverse changes in this area may have been caused not only by the lack of time to prepare healthy meals. Stress and anxiety associated with the pandemic, compounded by increased workload and the need for round-the-clock childcare, might have been other factors behind this change. Ammar et al. (2020) observed that emotional factors were associated with increased consumption of products with low nutritional values during the pandemic (especially sweets, with a 50% consumption increase) [23]. However, in the case of sweets and increased working hours, it was observed that after taking into account the sociodemographic factors, this relationship was on the verge of statistical significance (*p =* 0.05). It was the same with people who looked after a child 24 h a day and less frequently declared that they began to follow the principles of healthy eating during a pandemic. Therefore, it can be assumed that other factors such as gender, age, and place of residence could have influenced.

#### 4.2.4. Consumption Frequency for Selected Product Groups

The research did not identify significant changes in the frequency of fruit and vegetable consumption, although results from other studies showed that stress related to “natural disasters” did lead to a reduction in their consumption [19]. Such a trend was observed, among others, by Mitchell et al. (2020), who reported lower consumption of salads, vegetables, and fruit for a sample of 381,564 people [24]. A study by Deschasaux-Tanguy (2020) [21] yielded similar results. Different results were also observed for meat consumption frequency during the pandemic. Contrary to other studies that showed a reduction in its consumption (mainly due to difficulties in purchasing), there was an increase in meat consumption for students and remote workers. The results for fruit and vegetable as well as meat consumption differed compared to the studies cited. However, increased consumption was observed for products such as sweets and savory snacks, which was consistent with the results obtained by Cicero et al. (2020) [25]. The only group with lower consumption of such products compared to the other respondents comprised those whose life situations had not changed during the pandemic. This may indicate that the aforementioned emotional factors associated with increased consumption of such products were not only caused by the pandemic. Perhaps this indicates that a change in life situation compounded stress or feelings of anxiety.

#### 4.2.5. Physical Activity

Closure of gyms and swimming pools and limited opportunities to exercise at home (due to limited space, among other reasons) and outside of the house, as well as boredom and worse moods, may affect physical activity levels during the pandemic [26]. Ammar et al. (2020) observed reduced levels of physical activity in remote workers [23]. In our study, we also observed that these people reported reduced physical activity. However, it was not a statistically significant relationship (*p* > 0.05). On the other hand, the results of conducted studies indicated that the pandemic had a positive impact on physical activity in some groups. This was observed for students, who were more likely than others to declare increased physical activity (*p <* 0.01). However, the relationship between the level of physical activity and return to the family home was on the verge of statistical significance (*p =* 0.05) after taking into account sociodemographic factors. Therefore, it can be assumed that it was not the return to the family home but the gender or the place of residence that mattered here. Moreover, it was observed that increased physical activity in students did not translate into positive changes in eating behavior, although Romero-Blanco et al. (2020), in their study, observed that diet quality was better for students with higher levels of physical activity [27]. Studies provided different results by Ingram et al. (2020) and Gallo et al. (2020), who reported that students’ physical activity levels decreased during the pandemic [4,26].

### 4.3. Strengths and Limitations

The study aimed not only to identify changes in eating behaviors and physical activity during the pandemic but also to examine their relationship with changes in life situations at that time, which can be considered an advantage of the study. It seems relevant because a change in a life situation may involve emotional, financial, or other factors that further influence eating behaviors and physical activity. In addition, the study took into account six types of changes in living situations that may have affected eating behaviors and physical activity in different ways (e.g., due to financial factors for a decrease in business income or the amount of time for an increase/decrease in working hours). In comparison, Ingram et al. (2020), who also considered a change in life situation during the pandemic in their study, looked at two factors: change in work status and childcare [4]. Inclusion of the group whose life situation had not changed is also a strength of this study. It may be a benchmark and could help determine whether a change in life situation alone predisposes to specific changes in eating behavior and physical activity levels.

The research also has weaknesses, which include the subjective nature of responses. It was not conducted before and during the pandemic: Respondents were asked to self-compare their eating behaviors and physical activity levels for these two time periods. In addition, the research was web-based, and, therefore, despite providing instructions on how to complete the questionnaire correctly, some questions could have been misinterpreted.

Another weakness of the study is that it did not take into account sociodemographic factors, such as education, living conditions, or earnings. The primary limitation is that the convenience sampling strategy overrepresented certain categories, particularly women.

Another weakness of the study is that it lasted only a few months and was conducted in the early stages of the pandemic. Over time, there have been many changes, including the introduced restrictions (functioning of schools and universities, returning employees to offices, opening a gym, etc.) and the approach to this situation (we got used to some changes over time). Therefore, the presented results can only be applied to the situation at the beginning of the pandemic.

## 5. Conclusions

The results of the study indicated that a change in a life situation during the pandemic, such as a reduction in business income, decrease or increase in working hours, 24/7 childcare, a transition to remote working, and a return to the family home for students, can affect eating behaviors and physical activity levels. A variety of factors that result from a change in life situations may have a positive or a negative impact. These include emotional and financial aspects, as well as having more or less time to prepare healthy meals, eating out less often, or students returning to a family home.

Finding relationships between changes to life situations during the pandemic, eating behaviors, and physical activity helps identify groups that are particularly vulnerable to reduced diet quality and reduced levels of physical activity. This can help in planning activities that promote healthy lifestyles in these groups. The choice of their form may affect effectiveness. For example, students whose lifestyles have changed significantly during a pandemic are more likely to use social media. Therefore, through them, a healthy lifestyle can be promoted. On the other hand, in the case of people who, during a pandemic, have less time to follow the rules of a healthy lifestyle (e.g., due to 24-h childcare or increased working hours) a good solution can be mobile applications with healthy and simple recipes. The potential of modern technology may be used in order to promote solutions that help in adherence to the principles of healthy eating even in a small amount of time.

In this difficult time it is essential to identify the groups most exposed to negative changes and then introduce activities promoting the principles of healthy eating and physical activity during the pandemic, tailored to the needs of each group. A healthy diet and regular exercise can be immunomodulating. That is why adherence to a healthy lifestyle is so important in the pandemic.

## Figures and Tables

**Table 1:** General characteristics of the study population.

Variables	n (%)
N _total_	921 (100.00)
Country	
European countries	766 (83.17)
non-European countries	155 (16.83)
South America	60 (38.51)
North America	53 (34.16)
Australia	42 (27.33)
Age	
<35 years	589 (63.95)
>35 years	332 (36.05)
Gender	
female	734 (79.70)
male	187 (20.30)

**Table 2:** Change in life situation acc. to country, gender, and age.

	Reduced Business Income	24/7 Childcare	Transition to Remote Work	Increased Working Hours	Decreased Working Hours	Return to the Family Home (Student)	The Pandemic Had Not Affected Any Aspect of My Life
All Country	11.62% (n = 107)	11.29% (n = 104)	38.00% (n = 350)	15.20% (n = 140)	16.94% (n = 156)	14.44% (n = 133)	19.00% (n = 175)
European countries	10.05% ** (n = 77)	11.10% (n = 85)	39.16% (n = 300)	14.49% (n = 111)	16.06% (n = 123)	16.32% ** (n = 125)	19.32% (n = 148)
non-European countries	19.35 ** (n = 30)	12.26% (n = 19)	32.26% (n = 50)	18.71% (n = 29)	21.29% (n = 33)	5.16% ** (n = 8)	17.42% (n = 27)
Gender							
female	10.63% (n = 78)	12.67% * (n = 93)	38.15% (280)	15.12% (n = 111)	15.12% * (n = 111)	16.35% * (n = 120)	18.53% (n = 136)
male	15.51% (n = 29)	5.88 * (n = 11)	37.43% (n = 70)	15.51% (n = 29)	24.06 * (n = 45)	6.95% * (n = 13)	20.86% (n = 39)
Age							
<35 years	9.17% * (n = 54)	7.47% ** (n = 44)	42.78% ** (n = 252)	16.81% (n = 99)	15.45% (n = 91)	22.24% ** (n = 131)	15.62% ** (n = 92)
>35 years	16.01% * (n = 53)	18.13% ** (n = 60)	29.31% ** (n = 97)	12.39% (n = 41)	19.64% (n = 65)	0.60% ** (n = 2)	25.08% ** (n = 83)

* Significant differences, *p*-value < 0.05 ** Significant differences, *p*-value < 0.01.

**Table 3:** Changes to meal consumption regularity acc. to life situation during the pandemic.

	No Change	I Eat Meals Less Regularly	I Eat Meals More Regularly	*p*
reduced business income	51.40 % (n = 55)	14.95% (n = 16)	33.64% (n = 36)	>0.05
other respondents	45.82% (n = 373)	21.74% (n = 177)	32.43% (n = 264)	
24/7 childcare	44.23% (n = 46)	26.92% (n = 28)	28.85% (n = 30)	>0.05
other respondents	46.76% (n = 382)	20.20% (n = 165)	33.05% (n = 270)	
transition to remote work	39.43% (n = 138)	21.71% (n = 76)	38.86% (n = 136)	<0.01 *
other respondents	50.79% (n = 290)	20.49% (n = 117)	28.72% (n = 164)	
increased working hours	42.86% (n = 60)	27.14% (n = 38)	30.00% (n = 42)	>0.05
other respondents	47.12% (n = 368)	19.85% (n = 155)	33.03% (n = 258)	
decreased working hours	39.10% (n = 61)	17.95% (n = 28)	42.95% (n = 67)	<0.01 *
other respondents	47.97% (n = 367)	21.57% (n = 165)	30.46% (n = 233)	
return to the family home (students)	31.58% (n = 42)	21.80% (n = 29)	46.62% (n = 62)	<0.001 *
other respondents	48.89% (n = 386)	20.81% (n = 164)	30.20% (n = 238)	
the pandemic had not affected any aspect of my life	62.86% (n = 110)	18.86% (n = 33)	18.29% (n = 32)	
other respondents	42.63% (n = 318)	21.45% (n = 160)	35.92% (n = 268)	<0.001 *

* Significant differences.

**Table 4:** Changes to snacking acc. to life situation during the pandemic.

	No Change	I Stopped Snacking	I Started Snacking	*p*
reduced business income	44.86% (n = 48)	12.15% (n = 13)	42.99% (n = 46)	
other respondents	46.19% (n = 376)	15.48% (n = 126)	38.33% (n = 312)	>0.05
24/7 childcare	42.31% (n = 44)	14.42%(n = 15)	43.27% (n = 45)	
other respondents	46.51% (n = 380)	15.18% (n = 124)	38.31% (n = 313)	>0.05
transition to remote work	42.29% (n = 148)	17.14% (n = 60)	40.57% (n = 142)	
other respondents	48.34% (n = 276)	13.84% (n = 79)	37.83% (n = 216)	>0.05
increased working hours	42.14% (n = 59)	12.14% (n = 17)	45.71% (n = 64)	
other respondents	46.73% (n = 365)	15.62% (n = 122)	37.64% (n = 294)	>0.05
decreased working hours	46.79% (n = 73)	13.46% (n = 21)	39.74% (n = 62)	
other respondents	45.88% (n = 351)	15.42% (n = 118)	38.69% (n = 296)	>0.05
return to the family home (students)	32.33% (n = 43)	16.54% (n = 22)	51.13% (n = 68)	
other respondents	48.35% (n = 381)	14.85% (n = 117)	36.80% (n = 290)	*p <* 0.001 *
the pandemic had not affected any aspect of my life	61.71% (n = 108)	10.29% (n = 18)	28.00% (n = 49)	
other respondents	42.36% (n = 316)	16.22% (n = 121)	41.42% (n = 309)	<0.001 *

* Significant differences.

**Table 5:** Change in the level of physical activity acc. to life situation during the pandemic.

	The Level of Physical Activity Increased	The Level of Physical Activity Decreased	No Change	*p*
reduced business income	20.56% (n = 22)	52.34% (n = 56)	27.10% (n = 29)	>0.05
other respondents	21.99% (n = 179)	51.11% (n = 416)	26.90% (n = 219)	
24/7 childcare	13.46% (n = 14)	60.58% (n = 63)	25.96% (n = 27)	>0.05
other respondents	22.89% (n = 187)	50.06% (n = 409)	27.05% (n = 221)	
transition to remote work	22.86% (n = 80)	54.57% (n = 191)	22.57% (n = 79)	>0.05
other respondents	21.19% (n = 121)	49.21% (n = 281)	29.60% (n = 169)	
increased working hours	21.43% (n = 30)	50.00% (n = 70)	28.57% (n = 40)	>0.05
other respondents	21.90% (n = 171)	51.47% (n = 402)	26.63% (n = 208)	
decreased working hours	23.72% (n = 37)	51.28% (n = 80)	25.00% (n = 39)	>0.05
other respondents	21.44% (n = 164)	51.24% (n = 392)	27.32% (n = 209)	
return to the family home (students)	32.33% (n = 43)	48.12% (n = 64)	19.55% (n = 26)	<0.01 *
other respondents	20.05% (n = 158)	51.78% (n = 408)	28.17% (n = 222)	
the pandemic had not affected any aspect of my life	15.43% (n = 27)	47.43% (n = 83)	37.14% (n = 65)	
other respondents	23.32% (n = 174)	52.14% (n = 389)	24.53% (n = 183)	0.001 *

* Significant differences.

## Data Availability

Data can be accessible upon request to corresponding author.
